# Supplementary material for: Collaborative intelligence in AI: Evaluating the performance of a council of AIs on the USMLE
Source: PLOS Digit Health. 2025 Oct 9;4(10):e0000787. doi: 10.1371/journal.pdig.0000787 (PMC12510544; doi:10.1371/journal.pdig.0000787)
Supplement: S2 File — (ZIP) [file pdig.0000787.s002.zip › S2 Data Raw data files containing unprocessed question inputs and LLM outputs including transcripts of deliberations/STEP3_Question-32-1_Type-MC-NJ.docx]

**Facilitator** **[Round 1]:** A 32-year-old woman comes to the emergency department because of a 3-day history of fatigue and postural light- headedness. She also reports nausea with one episode of vomiting this morning and adds that her stools and urine have been darker than usual lately. Medical history is significant for stage III-B Hodgkin lymphoma. She started the first cycle of chemotherapy 8 days ago. Additional medications include ferrous gluconate, hydrocodone, and prochlorperazine. She is currently receiving 0.9% saline at 75 mL/hr. Vital signs today are temperature 37.5°C (99.5°F), pulse 76/min supine and 80/min standing, respirations 16/min, and blood pressure 125/65 mm Hg supine and 120/60 mm Hg standing. Pulse oximetry on room air shows an oxygen saturation of 94%. Lungs are clear to auscultation. Cardiac examination discloses a soft systolic murmur at the left upper sternal border. There is no hepatosplenomegaly or tenderness on abdominal examination. Rectal examination discloses no masses. Stool is black and test for occult blood is negative. Results of laboratory studies are shown: Blood Urine Hematocrit 24% Specific gravity 1.032 (N=1.010–1.025) WBC 2400/mm3 Bilirubin Negative Platelet count 78,000/mm3 RBC Negative Which of the following is the most likely explanation for the patient's current condition? (A) Adverse effect of medications (B) Bone marrow metastases (C) Gastrointestinal blood loss (D) Hemodilution (E) Hemolytic anemia

**AI_B:** (1) RESTATING THE QUESTION: The patient is a 32-year-old woman presenting with fatigue, light-headedness, nausea, vomiting, and unusually dark stools and urine. She has stage III-B Hodgkin lymphoma and started chemotherapy 8 days ago. Her current medications are ferrous gluconate, hydrocodone, and prochlorperazine. She's also receiving saline at a rate of 75mL/hr. Physical and laboratory examinations show anemia and normal stool occult blood. The query asks for the probable cause of her condition. (2) PLANNING: The assumptions are that the symptoms might be: - An adverse effect of the chemotherapy or other medications she's taking - A result of bone marrow metastases - Caused by gastrointestinal blood loss - Due to hemodilution - A result of hemolytic anemia. Each of these assumptions has potential validity, but we have to prioritize the most plausible one based on the patient's comorbidities, her lab results, and other clinical findings. (3) CLINICAL REASONING: Given the patient's medical history and her recent chemotherapy, it's reasonable to consider the possibilities of medication side effects or bone marrow metastases. However, her lab results provide clues that suggest otherwise; she presents with anemia and low white blood cell and platelet counts, reflective of a state of pancytopenia. This finding itself can occur due to bone marrow suppression, which could occur as a result of bone marrow metastasis or as an adverse effect of her chemotherapy, especially in Hodgkin's lymphoma. However, since her stool occult blood test is negative, it's less likely that her anemia is resultant from gastrointestinal blood loss. Moreover, despite her receiving saline her hematocrit level is still low, which does not really support hemodilution as the cause of her anemia. Additionally, the normal bilirubin in urine may argue against hemolytic anemia. (4) CONCLUDING: Based on her presentation, the absence of signs of gastrointestinal bleeding, the lack of evidence supporting hemodilution or hemolytic anemia, the hypothesis that prioritizes the effects of her recent chemotherapy regimen appears to be the most appropriate. (5) RESPONSE: Therefore, the most likely explanation for her current condition is (A) Adverse effect of medications, specifically, chemotherapy-related bone marrow suppression leading to pancytopenia.

**AI_D:** RESTATING THE QUESTION: This patient presents with several symptoms like fatigue, postural lightheadedness, darker stools and urine, and nausea with an episode of vomiting. She has a significant medical history of stage III-B Hodgkin lymphoma and just started chemotherapy 8 days ago. Her labs show a low hematocrit, low WBC, and low platelet count. We're asked to evaluate the most likely explanation for her current condition out of the options given: adverse effect of medications, bone marrow metastases, gastrointestinal blood loss, hemodilution, and hemolytic anemia. PLANNING: In this step, I will evaluate each assumption one by one. (A) Adverse effect of medications: This assumption is grounded on the patient's medication history, onde of which could cause her symptoms. (B) Bone marrow metastases: This assumption is based on the fact that she has a history of Hodgkin lymphoma, increasing the likelihood of metastases. (C) Gastrointestinal blood loss: An assumption could be made considering the patient's darker stool, but the occult blood test was negative. (D) Hemodilution: This assumption would involve the presence of diluting factors in the blood, causing the low levels of blood components. (E) Hemolytic anemia: This assumption is related to the low hematocrit, suggesting anemia, and the darker urine could be from the hemoglobin. CLINICAL REASONING: (A) Considering her medication history, they can indeed have side effects, but none of her current medications are known to significantly contribute to hematological changes observed. (B) Bone marrow metastases could indeed lead to a reduction in all cell lines, but metastasis is unusual in Hodgkin lymphoma, especially without other systemic symptoms. (C) Regarding gastrointestinal blood loss, she has black stools which might suggest melena, but the test for occult blood is negative and she does not present any other sign of gastrointestinal bleeding. (D) Hemodilution implies an increase in plasma volume which dilutes the blood cell counts. However, she is not presenting with symptoms compatible with fluid overload. (E) Hemolytic anemia can cause fatigue and lightheadedness due to the decrease in red blood cells. They can also lead to darker urine due to hemoglobinuria. In addition, her recent chemotherapy treatment could have resulted in hemolytic anemia due to its toxic effect on red blood cells. CONCLUDING: Based on all the reasoning above, the most probable explanation for the patient's current condition would be (E) hemolytic anemia, as a possible adverse effect of her recent chemotherapy treatment. Although other options were plausible, they were not strongly supported by the presented case. RESPONSE: (E) The most likely explanation for the patient's current presentation is Hemolytic anemia. The patient's low hematocrit, combined with her recent chemotherapy and appearance of darker urine, fit with this diagnosis.

**AI_C:** RESTATING THE QUESTION: The patient is a 32-year-old female with a history of stage III-B Hodgkin lymphoma, who has recently begun chemotherapy. In the last three days, she has been suffering from symptoms of fatigue and light-headedness when she changes her position. Furthermore, she is experiencing nausea and has had one episode of vomiting, and she has noticed darkening of her stool and urine. Her vital signs and physical examination are relatively normal, except for a soft systolic murmur at the left upper sternal border. Her most concerning lab findings are her low hematocrit (24%), low white blood cell count (WBC: 2400/mm3), and low platelet count (78,000/mm3). This presentation could be explained several ways, including adverse effects of medications, bone marrow metastases, blood loss in the digestive tract, dilution of blood constituents (hemodilution), or accelerated destruction of red blood cells (hemolytic anemia). PLANNING: Considering all the information gathered from the clinical history, symptoms reports, and lab results, certain assumptions can be made. The chemotherapy might have caused myelosuppression, leading to a decreased production of blood cells in the bone marrow. Alternately, the cancer could have metastasized to the bone marrow, interfering with normal blood cell production. Another possibility is gastrointestinal blood loss, but the test for occult blood was negative. Hemodilution could also occur and cause low blood values, but would not affect absolute cell numbers. Lastly, the symptoms could be associated with hemolytic anemia, an increased rate of red blood cell destruction. CLINICAL REASONING: In this case, the most likely cause of the patient's symptoms is related to her hematocrit, platelet, and WBC levels. She exhibits signs of pancytopenia (decreased RBCs, WBCs, and platelets). This finding is suggestive of bone marrow suppression, which could be due to either metastasis (Bone marrow metastases) or an adverse reaction to chemotherapy drugs (Adverse effect of medications). However, the metastasis of Hodgkin's lymphoma to bone marrow is a relatively rare occurrence in comparison to the myelosuppressive effects of chemotherapy drugs. Additionally, other options such as Hemodilution or Hemolytic anemia are less likely given the normal findings in hemoglobin evaluation, and lack of corroborative symptoms or supporting lab results, respectively. Despite dark stools (which might suggest Gastrointestinal blood loss), the occult blood test was negative, making this option less likely as well. CONCLUDING: Taking all of these factors into account, it is most likely that the patient's symptoms are a result of the myelosuppressive effects of her chemotherapy treatment. These drugs can decrease the activity of the bone marrow and result in a decrease in all blood cell types (pancytopenia), which would explain her presentation and lab findings. RESPONSE: Therefore, the most likely explanation for the patient's current condition is (A) Adverse effect of medications.

**AI_A:** I. RESTATING THE QUESTION: The question pertains to a 32-year-old female patient who is seeking medical assistance because she's experiencing fatigue, light-headedness, nausea, and has had one occurrence of vomiting. She notes that her stools and urine have been unusually dark. She's been diagnosed with stage III-B Hodgkin lymphoma and has recently commenced chemotherapy. Furthermore, she's on several medications, one of which includes ferrous gluconate, a form of iron used to treat iron deficiency anemia. Her current vitals indicate signs of mild dehydration and her lab results show a decrease in her hematocrit, white blood cell count, and platelet count. She's not displaying signs of internal bleeding as per her physical examinations, but her stools are dark even though her occult blood test is negative. Lastly, her urine specific gravity is high, signifying concentrated urine. We are asked to identify the most probable reason for her current condition considering the following options: adverse effect of medications, bone marrow metastases, gastrointestinal blood loss, hemodilution, or hemolytic anemia. II. PLANNING: Given that she's recently started chemotherapy, the drop in her hematocrit, WBC, and platelet count could be an adverse effect of the chemotherapy or due to bone marrow metastases. The increased urine specific gravity and the saline administration suggest dehydration, but this doesn't explain her low hematocrit count. Her darker stools and urine raise suspicion for blood loss, although her negative test for occult blood makes gastrointestinal blood loss less likely. Hemodilution could also account for the decreased hematocrit, but her high urine specific gravity points to concentration, not dilution. Lastly, hemolytic anemia might explain the low hematocrit and the dark urine, but there is typically an increase in bilirubin, which is not present in this case. Conversely, dark urine and stool could also be related to the ferrous gluconate she is consuming, which has not been considered in the options. III. CLINICAL REASONING: Starting with the adverse effects of medications, it's important to highlight that chemotherapy commonly causes myelosuppression leading to cytopenias, which could be the origin of the patient's low hematocrit, WBC count, and platelet count. Bone marrow metastasis is less likely in Hodgkin lymphoma, which typically stays within the lymphatic system and doesn't spread to the bone marrow. Thus, options (A) adverse effects of medication or (C) gastrointestinal blood loss would be more likely. While fatigued and light-headedness are symptoms of anemia, the gastrointestinal blood loss option seems less viable considering the negative fecal occult blood test and the absence of abdominal pain or tenderness, which would be typical symptoms of GI bleeding. Hemodilution might cause a drop in hematocrit, but doesn't explain the high urine specific gravity. Hemolytic anemia could explain the low hematocrit, but wouldn't necessarily present without a positive direct Coombs test or an increase in bilirubin level. IV. CONCLUDING: Weighing up the available evidence, the most plausible explanation considering the patient symptoms, her history of Hodgkin lymphoma and recent start of chemotherapy, and the laboratory results, is the adverse effects of medications; specifically, chemotherapy-induced myelosuppression. V. RESPONSE: Therefore, the most likely explanation for the patient's current condition is (A) Adverse effect of medications, particularly chemotherapy-induced myelosuppression.

**AI_E:** The question is asking what might be the primary cause of this 32-year-old woman's symptoms and laboratory findings. She has a history of stage III-B Hodgkin lymphoma and recently started chemotherapy. Her symptoms include fatigue, lightheadedness, nausea, vomiting, and changes in the color of her urine and stool. Some notable findings from physical examination and laboratory results are dark stool, a soft systolic murmur, hematocrit of 24%, white blood cell count of 2400/mm3, and platelet count of 78,000/mm3. The choices for the underlying cause include adverse effect of her current medications, bone marrow metastases, gastrointestinal blood loss, hemodilution, or hemolytic anemia. By restating the question, 'adverse effect of medications' would involve symptoms or abnormal lab results prompted by her current medications, 'bone marrow metastases' implies the spread of her lymphoma to the bone marrow, 'gastrointestinal blood loss' suggests blood loss in the digestive tract, 'hemodilution' means a decrease in the concentration of blood cells due to an increase in plasma volume, and 'hemolytic anemia' refers to the destruction of red blood cells leading to anemia. Given her clinical picture, all these could theoretically be relevant. Her medications might have induced some adverse actions, resulting in her symptoms. The chemotherapy she received for her Hodgkin lymphoma might have led to bone marrow metastases, leading to decreased production of different blood cells. It's also possible she's losing blood from her gastrointestinal tract, presenting as darker stools, or her blood cells could be getting diluted from receiving saline, or there might be an increased destruction of red blood cells causing anemia. But which one should we prioritize? We know from her symptoms, physical examination, and laboratory results that she has fatigue, lightheadedness, black stools, soft systolic murmur, low hematocrit, white blood cell count, and platelet count. These findings are suggestive of red blood cell destruction, decreased blood cell production, or loss of blood from the gastrointestinal tract. We can rule out 'adverse effect of medications' as her symptoms don't align with common side effects from her medications, and drug-induced cytopenia typically doesn't present with a change in stool color. We can also exclude 'hemodilution' as her being on 0.9% saline at 75 mL/hr is insufficient to cause such a substantial decrease in hematocrit, and her symptom of black stools can't be explained by hemodilution. Between 'bone marrow metastases', 'gastrointestinal blood loss', and 'hemolytic anemia', we'll start by considering bone marrow metastases. Though the patient's lymphoma could theoretically metastasize to the bone marrow leading to decreased production of blood cells, this doesn't account for the black stools. Furthermore, Hodgkin's lymphoma primarily involves the lymphatic system and rarely metastasizes to the bone marrow. Next, we'll evaluate 'gastrointestinal blood loss'. Not only does the patient's symptom of black stools suggest this, her decreased hematocrit, white blood cells, and platelet count could be due to losing these cells in her gastrointestinal tract. However, the test for occult blood was negative which makes this less likely. Finally, we'll consider 'hemolytic anemia'. This typically presents with paleness, fatigue, lightheadedness, and possibly darker urine due to the release of bilirubin upon the breakdown of red blood cells, all of which the patient has. Also, hemolytic anemia can cause an increased production of immature red blood cells which are larger than mature ones, leading to a decrease in hematocrit - a fitting explanation for our patient's lab results. Furthermore, her chemotherapy treatment could have potentially triggered this condition. In conclusion, when we connect these findings, the most likely explanation for the patient's current condition is 'hemolytic anemia', (E). Her fatigue, lightheadedness, nausea, vomiting, and changes in the color of her urine and stool, along with the laboratory findings, suggest this condition which could have been possibly triggered by chemotherapy for her Hodgkin lymphoma. This situation can exhaust the body's production capabilities leading to lower white blood cell and platelet counts.

**Facilitator** **[Round 2]:** Summary: AI_A, AI_B, AI_C, and AI_E all agree that the most likely explanation for the patient's condition is the adverse effect of medications, specifically chemotherapy-induced myelosuppression or bone marrow suppression leading to pancytopenia. They all rule out bone marrow metastases, gastrointestinal blood loss, hemodilution, and hemolytic anemia based on the patient's symptoms, lab results, and the nature of Hodgkin lymphoma. However, AI_D differs by suggesting that the most probable explanation for the patient's condition is hemolytic anemia, as a possible adverse effect of her recent chemotherapy treatment. AI_D argues that the patient's low hematocrit, combined with her recent chemotherapy and appearance of darker urine, fit with this diagnosis. Key Differences: The key difference in the perspectives lies in the interpretation of the patient's symptoms and lab results. AI_A, AI_B, AI_C, and AI_E believe that the patient's pancytopenia is most likely due to chemotherapy-induced myelosuppression, while AI_D believes that the patient's symptoms and lab results are more indicative of hemolytic anemia. Question for Council Members: Given the patient's symptoms, lab results, and recent chemotherapy treatment, what specific factors led you to your conclusion, and why do you believe these factors are more indicative of your chosen diagnosis (chemotherapy-induced myelosuppression or hemolytic anemia) over the other?. Based on your preceding response, how would you answer the original question: [A 32-year-old woman comes to the emergency department because of a 3-day history of fatigue and postural light- headedness. She also reports nausea with one episode of vomiting this morning and adds that her stools and urine have been darker than usual lately. Medical history is significant for stage III-B Hodgkin lymphoma. She started the first cycle of chemotherapy 8 days ago. Additional medications include ferrous gluconate, hydrocodone, and prochlorperazine. She is currently receiving 0.9% saline at 75 mL/hr. Vital signs today are temperature 37.5°C (99.5°F), pulse 76/min supine and 80/min standing, respirations 16/min, and blood pressure 125/65 mm Hg supine and 120/60 mm Hg standing. Pulse oximetry on room air shows an oxygen saturation of 94%. Lungs are clear to auscultation. Cardiac examination discloses a soft systolic murmur at the left upper sternal border. There is no hepatosplenomegaly or tenderness on abdominal examination. Rectal examination discloses no masses. Stool is black and test for occult blood is negative. Results of laboratory studies are shown: Blood Urine Hematocrit 24% Specific gravity 1.032 (N=1.010–1.025) WBC 2400/mm3 Bilirubin Negative Platelet count 78,000/mm3 RBC Negative Which of the following is the most likely explanation for the patient's current condition? (A) Adverse effect of medications (B) Bone marrow metastases (C) Gastrointestinal blood loss (D) Hemodilution (E) Hemolytic anemia]

**AI_E:** RESTATING THE QUESTION: The candidates for a 32-year-old female with Hodgkin lymphoma who is undergoing chemotherapy are experiencing unexplained fatigue, lightheadedness, darker stools, and urine, along with abnormal hematological and urinary lab results. The possible causes are: adversarial collateral effects from her medications (like significant myelosuppression), the spread of cancer to the bone marrow, bleeding in the gastrointestinal tract, hemodilution or a rise in plasma volume, and hemolytic anemia, a condition in which red blood cells are destroyed faster than they can be replaced. PLANNING: To ascertain the underlying cause, different assumptions related to the pathogenesis of these conditions must be taken into account. We determine this by her clinical presentation, her ongoing medical treatment, and her laboratory findings. Let's evaluate each assumption: An adverse effect of medications such as chemotherapy (myelosuppression) would present with pancytopenia, marrow metastases might bring forth similar signs, or if the metastases are exclusively involving red cell precursors, then isolated anemia might be there but this is quite rare. Gastrointestinal blood loss would likely present with a positive fecal occult blood test. Hemodilution usually occurs with volume overload and we don't have any evidence for such an occurrence. Hemolytic anemia, on the other hand, may present with symptoms of anemia, jaundice, and darker urine. CLINICAL REASONING: The patient's presentation with a decreased hematocrit, white blood cell count, and platelet count - i.e pancytopenia aligns with myelosuppression from chemotherapy, given also we know she started a chemotherapy cycle 8 days ago. High urine specific gravity and darker than usual urine & stool may suggest dehydration and may also indicate hemolysis. However, the absence of bilirubin in the urine rules out hemolysis as there would be bilirubinuria in hemolytic process. The negative fecal occult blood test makes the gastrointestinal bleed unlikely. Also, had there been bone marrow metastasis, the progression to pancytopenia within such a short span of time is unlikely, not typical for Hodgkin lymphoma. CONCLUDING: Based on her clinical presentation and the time course of her lab abnormalities, the most likely explanation for the patient's current condition would be adverse effects of medications, likely chemotherapy-induced myelosuppression. RESPONSE: So, to answer the original question, among the given options, the most likely explanation for this patient's current presentation is (A) Adverse effect of medications.

**AI_B:** Restating the question: AI_B needs to determine the most likely cause of a 32-year-old woman's presenting symptoms and findings, who is suffering from fatigue, postural lightheadedness, nausea, and changes in the color of her urine and feces. Her past medical history is significant as she has stage III-B Hodgkin lymphoma and has recently started her first cycle of chemotherapy. In terms of laboratory studies, she exhibits pancytopenia - a decrease in the number of red and white blood cells, as well as platelets. Planning: In answering this question, AI_B has laid out five potential causes for her condition: (A) Adverse effect of medications, (B) Bone marrow metastases, (C) Gastrointestinal blood loss, (D) Hemodilution, and (E) Hemolytic anemia. Each assumption has been generated based on her known history and current clinical presentation, but not all align with her symptoms and lab findings equally. Therefore, a prioritization needs to be determined clinically. Clinical Reasoning: Stage III-B Hodgkin Lymphoma suggests a widespread disease that has affected both sides of the diaphragm, a condition likely requiring aggressive chemotherapy. Given the timing, eight days post initiation of chemotherapy, it probably led to myelosuppression - a decrease in the production of blood cells in her body. This is reflected in her laboratory findings signify pancytopenia. Pancytopenia post chemotherapy would indicate an adverse effect of medications (Option A) more than it would suggest bone marrow metastases (Option B) which would not likely manifest this early. The black stool does hint at possible gastrointestinal blood loss (Option C), but the negative test for occult blood opposes this. Hemodilution (Option D) is not consistent as the patient is not overhydrated and this would generally raise all cell counts. Finally, nothing specific indicates that the patient suffers from hemolytic anemia (Option E), i.e., the destruction of red blood cells in the body, as opposed to a decrease in their production. Concluding: Considering the discussed factors, chemotherapy-induced myelosuppression or marrow suppression is most likely causing the patient's pancytopenia. The timing of her symptoms and their nature - decreased ability to transport oxygen (leading to fatigue and postural lightheadedness), impaired immunity (risk of infection), and reduced clot formation (risk of bleeding), all reflect in alignment with this explanation. Response: Therefore, the most probable explanation for the patient's condition is the adverse effect of her medications, particularly her recent chemotherapy (Option A). The suppressive effect of the cytotoxic drugs on the bone marrow function led to a reduction in her red and white blood cells and platelets, causing her symptoms.

**AI_D:** RESTATING THE QUESTION: A 32-year-old woman with stage III-B Hodgkin lymphoma who is currently undergoing chemotherapy presented to the ER with fatigue, postural lightheadedness, nausea, and one episode of vomiting. These complaints are quite recent, dating back to just three days ago. Additionally, she's noticed that her stool and urine have been darker than usual lately. She takes ferrous gluconate, hydrocodone, and prochlorperazine for her medical conditions. She's being hydrated with 0.9% saline at 75 mL per hour. Despite these clear symptoms, her vitals, while indicating a slight fever, appear normal. A recent lab indicates notably low values for hematocrit, WBC, and platelet count. Given these findings, the question to answer is, what's the most likely explanation for her current condition? PLANNING: We know that chemotherapy can result in bone marrow suppression (i.e., myelosuppression) because the therapy not only kills cancer cells but also affects bone marrow cells leading to decreased production of red blood cells, white blood cells, and platelets. This is indicated by her pancytopenia (namely, the low hematocrit representing decreased red blood cell count, low WBC count, and low platelet count). On the other hand, the dark urine and stool could point to hemolytic anemia where red blood cells are destroyed and removed from the bloodstream before their usual lifespan is up, resulting in hemoglobin being filtered by the kidneys and leaving a dark color. However, her stool occult blood test is negative. CLINICAL REASONING: The timing of onset of her symptoms aligns with the expected timeline of chemotherapy-induced myelosuppression, which typically happens within 7 to 10 days after chemotherapy. While red urine and black stool might suggest hemolysis and resultant hemoglobinuria, her bilirubin and RBC tests in the urine are negative, undermining the possibility of hemolytic anemia. In addition, Hemolytic anemia does not explain her low WBC and platelet counts, while chemotherapy-induced myelosuppression does. CONCLUDING: Weighing all these factors, it seems the most likely explanation is chemotherapy-induced myelosuppression that has led to a decrease in red blood cell, white blood cell, and platelet production. As a result, she has developed anemia indicated by her low hematocrit, leukopenia suggested by her low WBC count, and thrombocytopenia due to her low platelet count. Her current medications do not seem to play a significant role in this scenario. RESPONSE: In light of the presented information, the most likely explanation for the patient's current condition is an adverse effect of medications, specifically chemotherapy (option A).

**AI_C:** RESTATING THE QUESTION: The patient in question is a 32-year-old woman, experiencing fatigue, postural lightheadedness, nausea, vomiting, and changes in the color of her urine and stool. She has a known history of stage III-B Hodgkin lymphoma and began chemotherapy treatment 8 days ago. She's also been taking ferrous gluconate, hydrocodone, and prochlorperazine concurrently. She shows no signs of hepatosplenomegaly or abdominal tenderness and her stool tested negative for occult blood. However, her lab results indicate several abnormal findings, including a low hematocrit, low WBC count, low platelet count, and a high specific gravity in her urine. The question is seeking the most plausible explanation for her current state from the provided choices: adverse effects of medications, bone marrow metastases, gastrointestinal blood loss, hemodilution, and hemolytic anemia. PLANNING: We need to make several assumptions: 1) The darkening of the patient's urine and stool and her laboratory findings can be indicators of her overall health. 2) The patient's current symptoms and lab results may be related to her recently started chemotherapy or other medications. 3) The patient's lowered hematocrit, WBC, and platelet count could be a sign of systemic problems like anemia, infection, or bleeding. We should evaluate the provided choices in light of these assumptions. CLINICAL REASONING: The patient's symptoms and lab results provide important clues. Let's evaluate the choices: (A) Adverse effect of medications: Chemotherapy is known to suppress bone marrow, causing pancytopenia – a reduction in red cells (leading to decreased hematocrit), white cells (leading to a low WBC count), and platelets (leading to a low platelet count). Consequently, this can cause fatigue, lightheadedness, and an immunocompromised state. Additionally, certain medications can also cause changes in stool and urine color. (B) Bone marrow metastases: Typically, these are associated with solid tumors and are less common with Hodgkin lymphoma. Furthermore, severe bone pain often accompanies bone marrow metastases. (C) Gastrointestinal blood loss: This condition would typically show symptoms such as bloody stool, pain, and weight loss. The patient's stools are dark but not bloody. (D) Hemodilution: There are no indications that the patient has experienced fluid overload, which would lead to a dilution effect. (E) Hemolytic anemia: Although the patient exhibits signs of anemia, there are no lab findings indicative of hemolysis, such as elevated bilirubin levels or a high reticulocyte count. CONCLUDING: Based on clinical reasoning and the patient's symptom presentation, lab results, and recent chemo treatment, the most likely explanation is the adverse effect of medications, notably chemotherapy-induced bone marrow suppression leading to pancytopenia. RESPONSE: (A) Adverse effect of medications is the most likely explanation for the patient's current condition.

**AI_A:** To answer the question posed, let's rephrase the context and define some terms. A 32-year-old woman has recently received chemotherapy for her Hodgkin lymphoma. She currently experiences symptoms such as fatigue, nausea, vomiting, lightheadedness when changing position, and has noticed darkening of her urine and stool. Her blood and urine tests show that her hematocrit, platelet count, and WBC are lower than normal, while her urine specific gravity is higher than usual. Our key assumption in this case is that this set of symptoms and lab findings are all linked and that they are all a result of one primary cause rather than multiple coinciding issues. We need to determine whether each of the possible explanations could cause all of these symptoms and whether they map onto the observed laboratory results. Let's look at each option: (A) Adverse effect of medications: Chemotherapy is a potent medication with numerous side effects, including myelosuppression causing lower hematocrit, platelet count, and WBC count as seen in the patient. (B) Bone marrow metastases: Usually, there is an increased production of cells in metastases, which is not consistent with the patient's results. (C) Gastrointestinal blood loss: Despite the patient's dark stool, her test for occult blood was negative, which suggests that this is unlikely. (D) Hemodilution: This refers to the dilution of the blood, which would typically be suggested by a decrease in hematocrit and other cell counts, but it is not consistent with the patient's specific gravity. (E) Hemolytic anemia: While this would explain the patient's low hematocrit, it doesn't align entirely with the low increased WBC count and platelet count. Following the thought process outlined above, the assumption that should be clinically prioritized is that the adverse effect of chemotherapy (myelosuppression causing lower hematocrit, platelet count, and WBC count) is responsible for the patient's condition. As the chemotherapy targets rapidly dividing cells, it affects not only the Hodgkin lymphoma cells but also cells in the bone marrow, leading to a reduction in the production of all blood cells, as indicated in the low counts of hematocrit, WBC, and platelet cells. The symptom of dark urine could be an effect of the chemotherapy itself or due to the high specific gravity, raising the possibility of dehydration which is consistent with the symptoms experienced by the patient. In conclusion, given the complete set of observed symptoms, lab results, and the characteristic side effects of chemotherapy, the plausible explanation for the patient's condition is the adverse effect of medications (Option A). This conclusion adheres to the woman's symptoms and also aligns with her history of recent initiation of Chemotherapy, which is known to cause such side effects. Connectively, the signs, symptoms and lab results seem to implicate myelosuppression, a known side effect of Chemotherapy as the cause. This explains the lower counts in hematocrit, platelets, and WBC, often seen in patients undergoing chemotherapy. The high specific gravity of her urine points to a possible state of dehydration, which aligns with her symptoms of nausea, vomiting and could itself be an effect of the medication.

**Facilitator** The consensus among the AI responses is that the most likely explanation for the patient's current condition is the adverse effect of medications, specifically chemotherapy-induced myelosuppression. This conclusion is based on the patient's symptoms, her recent initiation of chemotherapy, and her laboratory findings. The patient's symptoms of fatigue, postural lightheadedness, nausea, vomiting, and changes in the color of her urine and stool, along with her abnormal lab results, are all considered in the context of her recent chemotherapy treatment. The timing of her symptoms aligns with the expected timeline of chemotherapy-induced myelosuppression, which typically occurs within 7 to 10 days after chemotherapy. The patient's lab results show pancytopenia, a condition characterized by a decrease in the number of red and white blood cells, as well as platelets. This aligns with the known side effects of chemotherapy, which not only targets cancer cells but also affects bone marrow cells, leading to a decrease in the production of these blood cells. The other potential causes for her condition, such as bone marrow metastases, gastrointestinal blood loss, hemodilution, and hemolytic anemia, were considered but found to be less likely based on her symptoms, lab results, and medical history. For instance, the negative test for occult blood in her stool makes gastrointestinal blood loss unlikely, and the absence of bilirubin in her urine rules out hemolytic anemia. In conclusion, the consensus is that the adverse effect of chemotherapy, specifically myelosuppression, is the most likely explanation for the patient's current condition. This conclusion is based on a comprehensive evaluation of the patient's symptoms, lab results, and recent medical history.
